# Effect of composite yeast culture on the jejunal barrier function, inflammatory response, and microbial community structure of laying hens during the late stage of egg production

**DOI:** 10.3389/fvets.2024.1524726

**Published:** 2024-12-18

**Authors:** Qifei Luo, Lan Yang, Bazarragchaa Tumenjargal, Shixiong Liu, Jiabin Ma, Jie Ning, Zichen Yun, Xiaojie Zhang, Yingga Wu, Yu Lu, Xiaoxuan Wu, Lijuan Wang, Shirchin Demberel, Dacheng Liu

**Affiliations:** ^1^College of Veterinary Medicine, Inner Mongolia Agricultural University, Hohhot, China; ^2^Key Laboratory of Clinical Diagnosis and Treatment Techniques for Animal Disease, Ministry of Agriculture, Hohhot, China; ^3^Laboratory of Young Animal Physiology and Pathology, Institute of Veterinary Medicine, Mongolian University of Life Sciences, Ulaanbaatar, Mongolia; ^4^Shenyang Fengmei Biotechnology Co., Ltd., Shenyang, China

**Keywords:** composite yeast culture, production performance, intestinal barrier, cytokines, intestinal microbiota

## Abstract

During the late laying period, the intestinal barrier of laying hens is susceptible to damage, resulting in enteric infections and even systemic inflammatory responses, posing a major challenge for the poultry industry. Therefore, it is crucial to investigate methods for addressing intestinal inflammation in late laying hens. In order to maximize the production potential of egg laying chickens, farmers usually use various feed additives to prevent damage to the intestinal barrier. Composite yeast cultures have shown advantages in broiler applications. This study aims to assess the impact of composite yeast culture (CYC) on the intestinal barrier function, inflammatory cytokines, and microbial community structure of Hy-Line Brown laying hens. A total of 160 healthy Hy-Line Brown hens, aged 58 weeks and of similar weight, were randomly assigned to two groups, with four replicates per group and 20 hens in each replicate. The control group was fed a basal diet (Con), while the experimental group was provided with a diet supplemented with 40 g/kg of composite yeast culture (CYC). The test period was 25 days. The results indicated that: compared to the control group, CYC significantly improved the egg production rates of hens during days 11–15, 16–20, and 21–25 (*p* < 0.05). CYC significantly enhanced the relative mRNA expression levels of occludin, claudin-1, zonula occludens-1 (ZO-1), and mucin 2 (Muc2) in the intestinal tract (*p* < 0.05), while reducing the relative expression levels of pro-inflammatory factors TNF-α and IFN-γ (*p* < 0.05), and increasing the levels of anti-inflammatory factors IL-4, IL-10, and TGF-β1 (*p* < 0.05). CYC significantly increased the abundance of *Bifidobacterium pseudocatenulatum* and *Faecalibacterium prausnitzii* in the intestine. These findings suggest that the composite yeast culture (CYC) can improve the structure of the intestinal microbial community. In conclusion, CYC may enhance egg production rates, reduce inflammatory responses, and strengthen intestinal barrier function by modulating the composition of the intestinal microbiota in late laying hens.

## 1 Introduction

As the rearing time of laying hens extends from 72 weeks to 100 weeks, the late laying period accounts for more than half of the laying cycle (1). In intensive poultry farming systems, the metabolic activity of hens during peak laying, coupled with various oxidative stresses, leads to a decline in intestinal barrier function as the hens age. This deterioration can result in gut-derived infections and even systemic inflammatory responses, subsequently causing a decrease in egg production and a reduction in their productive lifespan, resulting in significant economic losses (2, 3). Therefore, the production potential of hens in the late laying period largely depends on a healthy gastrointestinal tract, with a complete intestinal barrier being particularly crucial.

At the same time, issues such as the misuse of antibiotics and illegal additives in livestock farming have come to light (4). To ensure the safety of animal-derived food products, China implemented a comprehensive ban on the addition of antibiotics to animal feed in 2020. Consequently, to address these issues, various feed additives have been sought and adopted to prevent damage to the intestinal barrier in laying hens, thereby reducing the occurrence of chronic intestinal inflammation in hens during the late laying period (5, 6). In recent years, probiotics and their cultures have emerged as environmentally friendly and efficient growth promoters and immune modulators, demonstrating significant potential for positive impacts on animal health and human welfare, and are considered ideal alternatives to antibiotics (7).

It has been demonstrated that yeast cultures can improve the immune function of broiler chickens, enhance nutrient metabolism, and increase production performance (8). Furthermore, yeast culture (specifically, *Saccharomyces cerevisiae*) additives have been shown to improve nutrient utilization and meat quality in broilers, as well as to enhance the ecological environment (9, 10). Yeast is commonly used as a feed additive in the forms of live yeast, dried yeast, fermentation products, and components of yeast cell walls (11). Beta-glucans and mannan oligosaccharides, the primary components of yeast cell walls, have been shown to play a significant role in the antioxidant capacity and immune response in animals such as broilers, sheep, and calves (12–15). Additionally, it has been widely reported that yeasts can enhance food digestion and nutrient absorption in broiler chickens by modulating or optimizing the composition of the intestinal microbiota, thereby improving growth performance (16, 17).

Currently, numerous probiotic fermentation products for poultry have emerged, but their quality and efficacy vary significantly. The composite yeast culture (CYC) is a microbial fermentation preparation previously developed and commercialized by our research group. It is derived from two high-activity yeast strains isolated from naturally fermented milk in the Inner Mongolian grasslands, fermented with a specific medium using a specialized fermentation process (18). The main active components of CYC include beta-glucans, mannan oligosaccharides, peptides, amino acids, and organic acids. Preliminary studies have shown that this microbial ecological preparation promotes growth and improves immune function in sheep (18). Additionally, research has found that beta-glucans can enhance production performance, egg quality, and immune function in laying hens (19). Mannan interacts with transmembrane glycoprotein receptors that recognize glycosylated compounds, stimulating macrophages *in vivo* and promoting phagocytosis (20). Although many studies indicate that CYC is beneficial for host health, this has yet to be confirmed in laying hens during the late laying period. Therefore, this study aims to investigate the effects of CYC feeding on growth performance, intestinal barrier function, inflammatory responses, and the intestinal microbiota structure in Hy-Line Brown laying hens during the late laying period, with the goal of providing a theoretical basis for the clinical application of such composite yeast cultures in the poultry industry.

## 2 Materials and methods

### 2.1 Preparation of composite yeast culture

The composite yeast culture (CYC) is a product of previous research conducted by our group, containing five primary active components, with their minimum concentrations listed in Table 1. A strain of *Saccharomyces cerevisiae* (BC) and a strain of *Kluyveromyces marxianus* (XR4) from the microbial ecological preparation strain bank of the Veterinary College of Inner Mongolia Agricultural University were mixed in a 1:1 ratio with distilled water to create a microbial suspension with a concentration of 10^9^ CFU/mL. This suspension was inoculated at an 8% inoculum rate into a solid-state fermentation substrate, to which water was added to achieve a moisture content of 40%. The fermentation lasted for 72 h. The solid-state fermentation substrate was composed of corn, rice bran, cottonseed, wheat, and bran. The fermentation process was carried out in a laboratory environment, with the temperature of the substrate recorded every 3 h. When the fermentation time reached 24 h and the core temperature reached 40°C, the substrate was turned over and continued to ferment. After 72 h of fermentation, the composite yeast culture was dried and ground at low temperatures (45–50°C) and then packaged for use.

**Table 1:** Main components and concentrations of CYC.

Component (mg/kg)	Content
Beta-glucans	1,450
Mannan oligosaccharides	670
Peptides	1.3
Amino acids	152.7
Organic acids	1.48

### 2.2 Experimental design and feeding management

The basic diet was formulated based on the NRC (1994) guidelines, utilizing a corn-soybean meal diet. The composition and nutritional levels of the basic diet are presented in Table 2. Prior to the start of the experiment, strict disinfection of the housing environment was conducted, and during the trial, manure was cleaned and the chicken house was disinfected daily.

**Table 2:** Composition and nutritional levels of the basic diet (dry matter).

Ingredient (%)	Content	Nutritional level	Content
Corn	64.0	Metabolizable energy (MJ/kg)	11.88345
Soybean meal	25.0	Crude protein (%)	16.84
Limestone	8.5	Calcium (%)	3.5462
Premix	2.0	Phosphorus (%)	0.4337
Soybean oil	0.5	Lysine (%)	0.9764
Total	100	Methionine (%)	0.3502

^1Vitamin premix provided per kilogram of feed provides: vitamin A, 5000 IU; vitamin D3, 2,500 IU; vitamin K3, 2.5 mg; vitamin B1, 1 mg; vitamin B2, 2 mg; vitamin B6, 3 mg; vitamin B12, 0.005 mg; D-calcium pantothenate, 20 mg; folic acid, 0.3 mg; nicotinamide, 20 mg.^

^2^Mineral premix provided per kg of feed: iron (FeSO_4_·7H_2_O, 20.09% iron), 400 mg; Cu (CuSO_4_·5H_2_O, 25.45% Cu), 12 mg; manganese (MnSO_4_·H_2_O, 32.49% manganese), 90 mg; zinc (ZnO, 80.35% Zn), 180 mg; iodine (NaI, 84.68% iodine), 2.5 mg; selenium (NaSeO_3_, 45.56% selenium), 0.5 mg.

This feeding trial was conducted at the Hy-Line Brown laying hen farm of Fengmei Biotechnology Co., Ltd. in Shenyang, Liaoning Province. A total of 160 healthy Hy-Line Brown laying hens, approximately 58 weeks old and of similar body weight, were randomly assigned to two experimental groups, each consisting of four replicates of 20 hens. The control group was fed a basic diet, while the experimental group received a diet supplemented with CYC (40 g/kg), with the supplementation level determined based on previous research conducted by our group on composite yeast cultures. The pre-feeding period lasted for 3 days, followed by a 25 day trial period. The hens were housed in four-tier stepped cages with three hens per cage, and automatic waterers were available. Feed three times a day (at 04:00, 11:00, and 17:00), and during the trial period, they can eat as they please. A 16-h photoperiod was maintained with natural lighting supplemented by artificial light controlled by a lighting program controller. The henhouse temperature was kept at 22 ± 2°C, utilizing natural ventilation along with longitudinal negative pressure ventilation.

### 2.3 Production performance

During the experiment, eggs were collected and weighed from each replicate daily at 9:00 AM. Additionally, leftover feed was collected and weighed every 3 days. The calculations were performed over a 5-day cycle, and the following metrics were calculated: egg production rate, average egg weight, average feed intake, and feed-to-egg ratio.


$$\text{Egg}\;\text{production rate}=\left(\begin{array}{l} \text{Number of eggs produced}/\\ \text{Number of hens} \end{array}\right)\times 100\%$$

Averageeggweight=Totaleggweight/Number of eggs

$$\text{Average feed intake}=\left(\begin{array}{l} \text{Total feed amount}-\\ \text{Remaining feed amount} \end{array}\right)/\text{Number hens}$$

Feed toeggratio=Averageeggweight/Average feed intake

### 2.4 Sample collection

At the end of the feeding period, 8 chickens from each group (2 chickens per replicate) were randomly selected. They were rendered unconscious via CO_2_ intravenous injection, followed by euthanasia through cervical dislocation. Subsequently, laboratory personnel wearing sterile gloves performed dissections. Approximately 10 g of intestinal content from the jejunum was collected using sterilized sampling tools and immediately transferred to a 2 mL sterile, enzyme-free cryogenic tube. A 2 cm segment of the jejunum was also collected under sterile conditions and transferred to another 2 mL sterile, enzyme-free cryogenic tube. The collected intestinal tissue and contents were rapidly frozen in liquid nitrogen and stored at −80°C for subsequent assessments of tight junction proteins, inflammatory cytokines, and metagenomic analysis. Additionally, intestinal tissue samples were collected for later histological analysis.

### 2.5 Histological analysis

For each hen, a 2 cm segment of the jejunum was collected, and the intestinal contents were carefully washed away using sterile PBS solution. Excess moisture was removed with filter paper, and the tissue was then fixed in 4% paraformaldehyde solution (Biosharp, Hefei, China) for at least 48 h. The samples were subsequently embedded in paraffin, and sections of 4 μm thickness were prepared using a microtome. Hematoxylin and eosin (HE) staining was performed on the tissue sections. Finally, the samples were observed under an optical microscope (NiKon DS-Fi3, Tokyo, Japan) at 40× magnification, and the heights of the intestinal villi (VH), crypt depths (CD), and the ratio of villus height to crypt depth (VH/CD) were measured and calculated using an image processing and analysis system (NIS-Elements F 5.21.00).

### 2.6 Gene expression measurement

Total RNA was extracted from the jejunal samples using the Total RNA Mini Kit (GeneBetter, Beijing, China). The quality and concentration of the obtained total RNA were measured using a microplate reader (BioTek Synergy HT, Richmond, United States). Using HiScript III RT SuperMix (Vazyme, Nanjing, China), 1 ng of total RNA was reverse transcribed to synthesize complementary DNA (cDNA).

Amplification was performed using the CFX96 Touch^™^ Real-time PCR System (Bio-Rad, California, United States) with SYBR Green (Biosharp, Hefei, China), ddH₂O, cDNA, and both forward and reverse primers. The entire RT-qPCR process was monitored to obtain the cycle threshold (Ct) values, and the expression levels of target genes were calculated using the $2^{-\Delta \Delta C_{t}}$ method. The primer sequences for all genes (β-actin, claudin-1, occludin, ZO-1, Muc2, IL-1β, TNF-α, IFN-γ, IL-4, IL-10, TGF-β1) can be found in Table 3. The reaction system consisted of 10 μL, and the program included an initial denaturation step (95°C, 2 min), followed by 40 cycles of denaturation (95°C, 15 s) and annealing/extension (60°C, 30 s). The entire experimental procedure was conducted according to the manufacturer’s instructions.

**Table 3:** Primer sequence.

Gene	Primer sequence (5′ to 3′)	GenBank ID PCR	Product (bp)
β-actin	Forward: TCCCTGGAGAAGAGCTATGAA	NM_205518.1	113
	Reverse: CAGGCTCCATACCCAAGAAAG		
Claudin-1	Forward: GATCCAGTGCAAGGTGTACG	NM_001013611.2	200
	Reverse: TGATGAAGATCACGCCACCA		
Occludin	Forward: GAGTTGGATGAGTCCCAGTATGAG	NM_205128.1	204
	Reverse: ATTGAGGCGGTCGTTGATG		
ZO-1	Forward: GGAGGATCCAGCCATGAAAC	XM_015278981.2	236
	Reverse: CTTGAGGTCTCTGTGGTTCTGG		
Muc2	Forward: TCAAGCCATCTTCTTACTACCTCA	XM_040701667.1	119
	Reverse: TTGAATGTCCTGGACTGACTGAT		
IL-1β	Forward: TACATGTCGTGTGTGATGAGCG	NM_204524.2	224
	Reverse: TGGTCGGGTTGGTTGGTGAT		
TNF-α	Forward: TTGACTTGGCTGTCGTGTGG	NM_204267.1	136
	Reverse: TATAAGAACCAACGTGGGCATT		
IFN-γ	Forward: AAGCTCCCGATGAACGACTTG	NM_205149.2	125
	Reverse: TTGCATCTCCTCTGAGACTGGC		
IL-4	Forward: CCAGCACTGCCACAAGAACC	NM_001007079.2	163
	Reverse: AGCTAGTTGGTGGAAGAAGGTACG		
IL-10	Forward: CGCTTCTACACAGATGAGGTCC	NM_001004414.3	176
	Reverse: AACGTCTCCTTGATCTGCTTGA		
TGF-β1	Forward: GCCGACACGCAGTACACCAA	NM_001318456.1	160
	Reverse: GGACCACCATATTGGAGAGCTG		

### 2.7 Metagenome sequencing

DNA extraction from the jejunal contents was carried out by Magergene (Guangdong Magigene Biotechnology Co., Ltd., Guangzhou, China) using a commercial kit in accordance with the manufacturer’s instructions. The integrity and purity of the extracted DNA were assessed using 1% agarose gel electrophoresis. Additionally, the concentration and purity of the DNA were measured using a Qubit 3.0 (Thermo Fisher Scientific, Waltham, United States) and a NanoDrop One (Thermo Fisher Scientific, Waltham, United States).

Following the manufacturer’s recommendations, the ALFA-SEQ DNA Library Prep Kit was used to generate sequencing libraries, including the addition of index codes. The quality of the libraries was assessed using the Qubit 4.0 fluorometer (Life Technologies, Grand Island, NY) and the QSEP400 high-throughput nucleic acid analysis system (Houze Biology Technology Co., China). Finally, the libraries were sequenced on the Illumina NovaSeq 6000 platform, yielding 150 bp paired-end reads for subsequent analysis. Use QIIME software to evaluate the alpha diversity index and display the results in a box plot. Use R software to draw PCoA and abundance maps.

### 2.8 Statistical analysis

GraphPad Prism 9.5.1 (GraphPad Software, Inc., San Diego, CA, United States) was used to analyze the data. Independent samples t-tests were conducted to compare the differences between groups. If the data are normally distributed but have heterogeneous variances, Welch’s correction *t*-test was employed. For non-normally distributed data, the Mann–Whitney *U* test, a non-parametric test, was utilized. The Spearman statistical method was used to analyze the correlation coefficients between the gut microbiota and the indicators of gut barrier function and inflammatory cytokines. Moreover, a *p* < 0.05 was considered statistically significant.

## 3 Results

### 3.1 Production performance

Based on the results presented in Table 4, there were no significant differences in average daily feed intake and feed-to-egg ratio between the two groups during the experimental phases (days 1–5, 6–10, 11–15, 16–20, and 21–25) (*p >* 0.05). However, during the periods of days 11–15, 16–20, and 21–25, the egg production rates of the CYC group were higher than those of the control group by 4.75, 6.25, and 7%, respectively, with significant differences (*p* < 0.05). In the period from days 6–10, the average egg weight of the CYC group was 0.49 g heavier than that of the control group, which was also statistically significant (*p* < 0.05). Additionally, the CYC group showed an increasing trend in average egg weight compared to the control group during the periods of days 11–15, 16–20, and 21–25, although these differences were not statistically significant (*p >* 0.05).

**Table 4:** Effect of CYC on performance of laying hens in the late laying period.

Items	Time	Con	CYC	SEM	*p*-value
Egg production rate (%)	Day 1 to 5	80.50	83.25	2.99	0.394
	Day 6 to 10	81.75	85.50	2.70	0.215
	Day 11 to 15	80.75^a^	85.50^b^	1.91	0.047
	Day 16 to 20	80.00^a^	86.25^b^	1.38	0.004
	Day 21 to 25	81.00^a^	88.00^b^	1.41	0.003
Average egg weight (g)	Day 1 to 5	60.42	60.68	0.15	0.132
	Day 6 to 10	60.55^a^	61.04^b^	0.17	0.029
	Day 11 to 15	61.12	61.39	0.20	0.204
	Day 16 to 20	61.67	61.72	0.16	0.769
	Day 21 to 25	61.51	61.58	0.17	0.699
Average feed intake (g)	Day 1 to 5	119.3	120.8	1.76	0.408
	Day 6 to 10	123.8	126.4	1.76	0.187
	Day 11 to 15	122.0	125.9	1.76	0.066
	Day 16 to 20	117.0	120.1	1.76	0.133
	Day 21 to 25	122.9	124.2	1.76	0.504
Feed to egg ratio	Day 1 to 5	1.97	1.99	0.026	0.524
	Day 6 to 10	2.05	2.07	0.024	0.309
	Day 11 to 15	2.00	2.05	0.024	0.063
	Day 16 to 20	1.90	1.95	0.026	0.116
	Day 21 to 25	2.00	2.03	0.027	0.336

^1^Data within the same row that lack letters indicate no significant difference (*p >* 0.05), while different letters indicate a significant difference (*p* < 0.05).

Overall, the inclusion of CYC in the diet of laying hens during the late egg-laying period positively influences egg production rates and average egg weight without adversely affecting feed intake or feed efficiency. These findings suggest that CYC could be a beneficial supplement for improving the production performance of laying hens during this critical phase.

### 3.2 Investigation results on the barrier function of the jejunum

**Figure 1:**
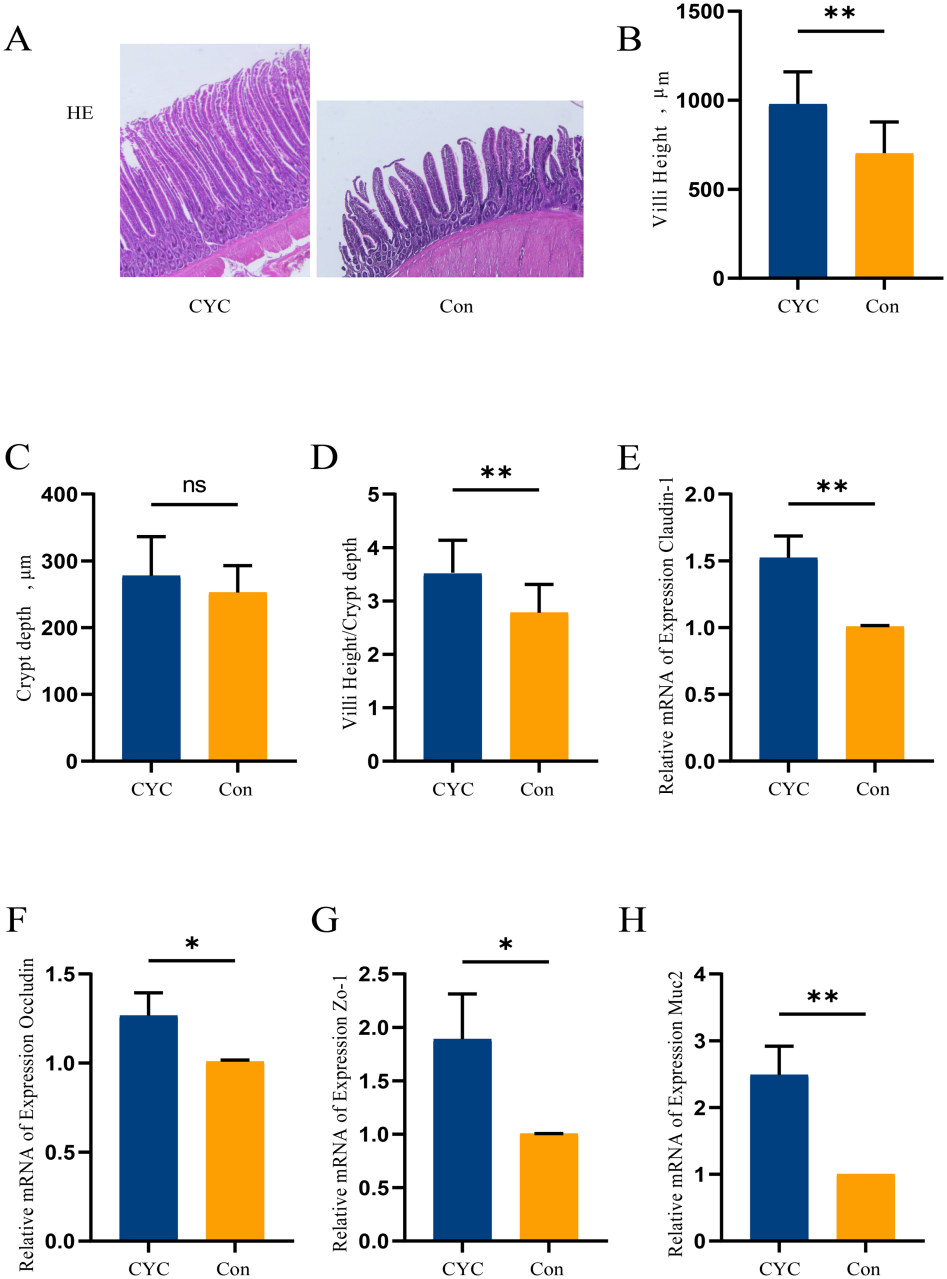
The impact of adding CYC on the jejunal barrier in laying hens during the late egg-laying period. **(A)** Observation of jejunal tissue morphology under 40× magnification using HE staining. **(B)** Measurement and calculation of jejunal villus height (VH). **(C)** Measurement and calculation of jejunal crypt depth (CD). **(D)** Calculation of the ratio of jejunal villus height to crypt depth (VII/CD). **(E)** Relative expression level of claudin-1 mRNA in jejunal tissue. **(F)** Relative expression level of occludin mRNA. **(G)** Relative expression level of zonula occludens-1 (ZO-1) mRNA in jejunal tissue. **(H)** Relative expression level of mucin 2 (Muc2) mRNA in jejunal tissue. ^*^p < 0.05 and ^**^p < 0.01.

HE staining of jejunal tissue was performed (Figure 1A). The results showed that the villi in the Con group were relatively loosely arranged, while the villi in the CYC group were closely packed and exhibited relatively intact structures. The height of the jejunal villi in the CYC group (Figure 1B) and the ratio of villus height to crypt depth (Figure 1D) were significantly higher than those in the Con group (*p* < 0.01). There was no significant difference in crypt depth between the two groups (Figure 1C) (*p* > 0.05).

According to the qPCR results, the mRNA expression levels of claudin-1 (Figure 1E) and Muc2 (Figure 1H) in the jejunal tissue of the CYC group were significantly higher than those in the Con group (*p* < 0.01). Additionally, the mRNA expression levels of occludin (Figure 1F) and ZO-1 (Figure 1G) in the CYC group were also significantly higher than those in the Con group (*p* < 0.05). Taken together, these findings suggest that feeding CYC may improve the structure of intestinal villi and enhance the intestinal barrier function in laying hens.

### 3.3 I results of the study on inflammatory cytokines in jejunal tissue

**Figure 2:**
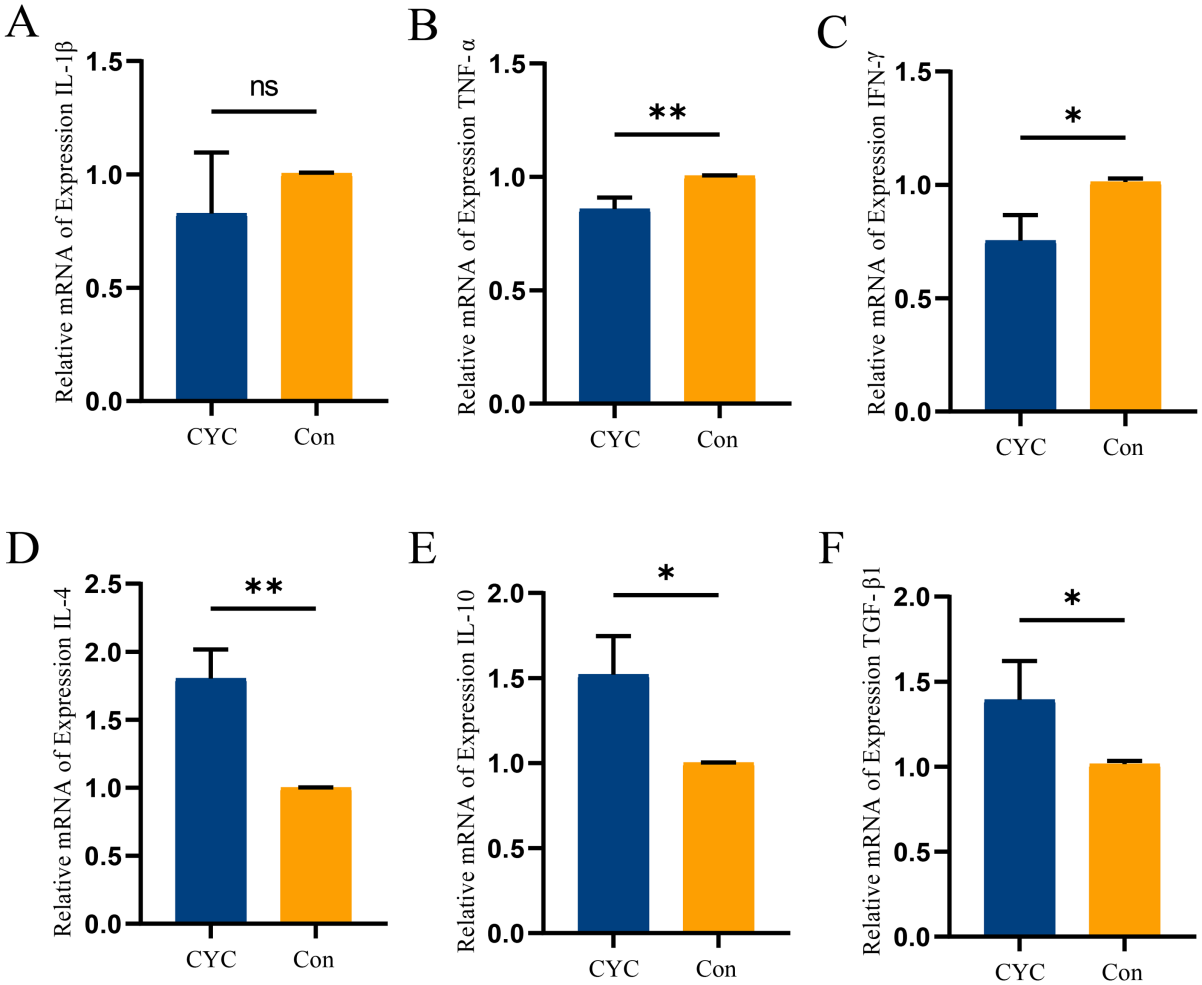
The impact of CYC on the expression of cytokine genes in the jejunum of laying hens during the late laying period. **(A)** Relative expression levels of mRNA for IL-1β in jejunal tissue. **(B)** Relative expression levels of mRNA for TNF-α in jejunal tissue. **(C)** Relative expression levels of mRNA for IFN-γ in jejunal tissue. **(D)** Relative expression levels of mRNA for IL-4 in jejunal tissue. **(E)** Relative expression levels of mRNA for IL-10 in jejunal tissue. **(F)** Relative expression levels of mRNA for TGF-β1 in jejunal tissue. ^*^p < 0.05 and ^**^p < 0.01.

Results of gene expression of inflammatory cytokines (Figure 2). The study found that the relative expression level of the pro-inflammatory cytokine TNF-α (Figure 2B) in the jejunal tissue of the CYC group was significantly lower than that of the Con group (*p* < 0.01). Additionally, the relative expression level of IFN-γ (Figure 2C) in the CYC group was significantly lower than that in the Con group (*p* < 0.05). There was a trend towards decreased expression of IL-1β (Figure 2A) in the CYC group, although this did not reach statistical significance (*p* > 0.05). Conversely, the relative expression level of the anti-inflammatory cytokine IL-4 (Figure 2D) in the CYC group was significantly higher than that of the Con group (*p* < 0.01). The relative expression levels of IL-10 (Figure 2E) and TGF-β1 (Figure 2F) in the CYC group were also significantly higher than those in the Con group (*p* < 0.05). In summary, feeding CYC to laying hens may primarily modulate intestinal inflammatory responses by reducing levels of IFN-γ and TNF-α, while enhancing levels of IL-4, IL-10, and TGF-β1, thereby influencing immune function.

### 3.4 Metagenomic analysis

**Figure 3:**
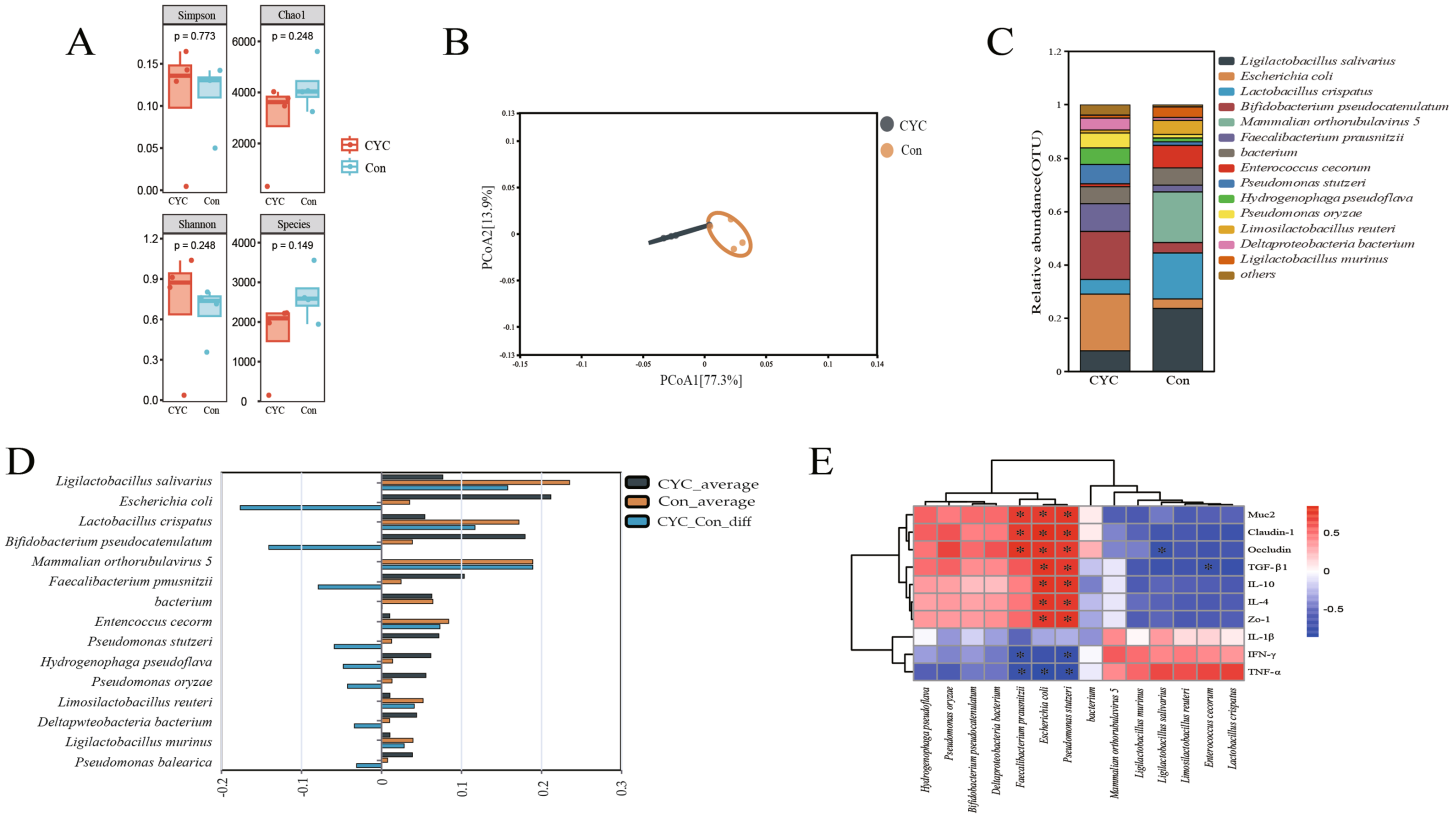
Effect of CYC supplementation on microbial structure in the jejunum during the late laying period. **(A)** α-diversity analysis. **(B)** Principal coordinate analysis (PCoA). **(C)** Bar chart of the relative abundance of the top 15 species at the species level. **(D)** Analysis of difference between two groups at different levels. **(E)** Heatmap of the correlation between the microbiota and relevant physiological indicators, where red indicates positive correlations and blue indicates negative correlations. ^*^p < 0.05.

We investigated the jejunal microbial structure of laying hens in the Con and CYC groups during the late laying period. The alpha diversity of microbial communities (Figure 3A) showed that there was no significant difference (*p* > 0.05) in Chao1, Shannon, Simpson, and Species indices between the CYC group and the Con group. The results of the β-diversity principal coordinate analysis (Figure 3B) indicated a significant separation distance between the Con and CYC groups, with a more pronounced intra-group clustering, suggesting a distinct difference in microbial composition between the two groups. At the species level, the composition results (Figures 3C,D) revealed that the abundances of *Escherichia coli*, *Bifidobacterium pseudocatenulatum*, *Faecalibacterium prausnitzii*, *Pseudomonas stutzeri*, *Hydrogenophaga pseudoflava*, *Pseudomonas oryzae*, and *Deltaproteobacteria bacterium* were significantly increased in the CYC group. In contrast, *Ligilactobacillus salivarius*, *Lactobacillus crispatus*, *Enterococcus cecorum*, *Limosilactobacillus reuteri*, and *Ligilactobacillus murinus* showed higher abundances in the Con group.

To further investigate the impact of the microbial composition in the jejunal tract on gut barrier function and inflammatory cytokines in laying hens during the late laying period, this study employed Spearman correlation analysis to assess the relationships between the top 14 most abundant microbial species and relevant physiological indicators. The correlation analysis results for the intestinal microbial structure of laying hens during the late laying period (Figure 3E) revealed that *Faecalibacterium prausnitzii* exhibited a significant positive correlation with Muc2, occludin, and claudin-1 (*p* < 0.05), while showing a significant negative correlation with IFN-γ and TNF-α (*p* < 0.05). Additionally, *Escherichia coli* was significantly positively correlated with Muc2, claudin-1, occludin, TGF-β1, IL-10, IL-4, and ZO-1 (*p* < 0.05) and negatively correlated with TNF-α (*p* < 0.05). *Pseudomonas stutzeri* displayed significant positive correlations with Muc2, claudin-1, occludin, TGF-β1, IL-10, IL-4, and ZO-1 (*p* < 0.05), alongside significant negative correlations with IFN-γ and TNF-α (*p* < 0.05). Lastly, *Ligilactobacillus salivarius* showed a significant negative correlation with occludin (*p* < 0.05), while *Enterococcus cecorum* and *Lactobacillus crispatus* were both significantly negatively correlated with TGF-β1 (*p* < 0.05).

In summary, the addition of CYC not only altered the microbial diversity in the jejunal tract of laying hens but also enriched beneficial bacterial species, which could contribute to enhanced gut health and improved gut barrier function during the late laying period.

## 4 Discussion

In poultry, digestion and absorption primarily occur in the jejunum, a process influenced by the intestinal morphology and enzyme activity (21). In this study, we assessed the effects of composite yeast culture (CYC) as a feed additive on growth and intestinal health-related parameters in late-stage laying Hy-Line Brown hens, including growth performance, intestinal barrier function, cytokine levels, and gut microbial structure. Our findings demonstrate that the feed additive CYC can serve as a suitable alternative to antibiotics, reducing intestinal-related inflammation in late laying hens.

Due to the intense metabolic activity experienced by laying hens during peak egg production, the metabolic level gradually declines in hens during the later stages of egg-laying, leading to a decrease in egg production capacity (22). This study found that hens fed CYC exhibited a significant increase in egg production rate starting from day 11 (*p* < 0.05). This result may be associated with the presence of β-glucans and mannans in CYC, which can enhance intestinal morphology and barrier function in poultry, thereby promoting digestion and absorption (23). Furthermore, probiotics may compete with harmful bacteria for adhesion sites and nutrients on the intestinal mucosa (24, 25), potentially inhibiting the growth of pathogenic bacteria, protecting gut health, and consequently improving protein utilization and egg production rates. Previous studies have confirmed the positive effects of various yeast strains on improving poultry production performance; for example, yeast cultures can significantly enhance average daily weight gain and feed efficiency in broilers (26), and the addition of β-glucans to feed can improve egg production rates in heat-stressed hens (19). In earlier research, incorporating yeast cultures into feed significantly increased average daily feed intake and reduced feed-to-weight ratio in sheep (18). In this experiment, no significant differences were observed in average daily feed intake and feed-to-egg ratio between the two groups, suggesting that the appetite-stimulating effects of yeast cultures may vary among different animal species.

The jejunum is a crucial organ for digestion and nutrient absorption in poultry, where its structural and functional integrity is essential not only for nutrient assimilation but also for reducing the invasion of external pathogenic microorganisms (27, 28). The villus height to crypt depth ratio (VH/CD) is a key indicator that reflects the digestive and absorptive capacity of the small intestine; a higher ratio indicates a more effective intestinal barrier function (29, 30). Research has shown that feeding yeast and other probiotics significantly increases the jejunal villus height in late laying hens (31). In this study, CYC was found to enhance jejunal villus height (*p* < 0.05), which supports improved digestive and absorptive capabilities, thereby promoting animal health. Furthermore, the CYC group exhibited a significant increase in the VH/CD ratio, consistent with previous findings.

The intestinal barrier is primarily composed of intestinal epithelial cells and tight junction complexes, serving as the structural basis for maintaining epithelial permeability and barrier function. An intact intestinal barrier is essential not only for nutrient absorption but also for reducing the invasion of external pathogenic microorganisms (32, 33). The tight junctions between intestinal epithelial cells mainly consist of claudin-1, occludin, ZO-1, and junctional adhesion molecules (JAM). Claudin-1 and occludin are key proteins in the transmembrane protein family, with claudin-1 playing a critical role in stabilizing intestinal permeability (34), while occludin primarily maintains tight junction stability (35). ZO-1 supports and maintains tight junction structure, participating in the linkage of transmembrane proteins, substance transport, and signal transduction (36). In our study, CYC was shown to enhance the mRNA expression of tight junction proteins occludin, claudin-1, ZO-1, and Muc2 in late laying hens (*p* < 0.05), thereby strengthening intestinal barrier function. Muc2 has been found to assist probiotics in colonizing the gut (37). Furthermore, yeast culture has significantly increased the expression of Muc2 in broiler ileum (38), and microbial preparations from yeast cell walls have raised the expression of tight junction protein genes in broilers (39). The combined application of β-glucans and mannans has been shown to improve gut barrier function in broilers (23), and these findings align with our results. This phenomenon further suggests that CYC’s enhancement of egg production may be related to the ability of β-glucans and mannans to improve intestinal barrier function in late laying hens, facilitating digestion and absorption, and consequently enhancing protein utilization.

Poultry lack lymph nodes and instead rely on dispersed lymphoid tissues, making the gut a crucial organ for immune function (40). Intestinal immune regulation primarily depends on cytokines secreted by immune cells. Based on this, we measured changes in the mRNA expression of pro-inflammatory cytokines IL-1β, TNF-α, and IFN-γ, as well as anti-inflammatory cytokines IL-4, IL-10, and TGF-β1, aiming to reflect the health status of the hens through these expression changes. Our study revealed that after CYC supplementation, late laying hens showed reduced levels of the pro-inflammatory cytokines TNF-α and IFN-γ (*p* < 0.05), while the levels of anti-inflammatory cytokines IL-4, IL-10, and TGF-β1 increased (*p* < 0.05). That elevated expression of pro-inflammatory cytokines IL-1β, TNF-α, and IFN-γ can inhibit the expression of tight junction proteins occludin and claudin-1, which leads to increased intestinal permeability and damage to barrier function (41). Therefore, in this study, the promotion of occludin and claudin-1 expression by CYC may be related to the reduced expression of IL-1β, TNF-α, and IFN-γ. Pro-inflammatory cytokine IFN-γ and anti-inflammatory cytokines IL-4 and IL-10 are secreted by Th1 and Th2 cells, respectively, and play significant roles in the regulation of intestinal tight junctions and the maintenance of epithelial barrier integrity (42). These cytokines are essential components of cellular immunity (43). TGF-β1 can inhibit inflammation in the gut by directly acting on immune cells, thereby modulating intestinal immune function (44). Additionally, the anti-inflammatory cytokine IL-10 can enhance immune function by reducing the expression of the pro-inflammatory cytokine IL-1β (45). Therefore, this study suggests that CYC may improve intestinal barrier function in late laying hens by regulating the expression of pro-inflammatory cytokines TNF-α and IFN-γ, as well as anti-inflammatory cytokines IL-4, IL-10, and TGF-β1. Furthermore, our research group has confirmed at the cellular gene and protein levels that β-glucans and mannans can activate the TLR4-mediated NF-κB/MAPK signaling pathway, leading to a reduction in the expression of pro-inflammatory cytokines IL-1β and TNF-α and an increase in anti-inflammatory cytokine IL-10 expression, thereby modulating cellular inflammatory responses. In summary, this study demonstrates that CYC supplementation effectively alleviates intestinal inflammation and enhances immune function in late laying hens.

Maintaining the microbial ecological balance in the gastrointestinal tract is crucial for overall health. Dominant microbial communities play an important role in achieving this balance (46). Numerous studies have demonstrated that gut microbiota can form a microbial barrier, participate in protecting the host’s intestinal defenses and nutrition, and regulate immune function (47, 48). In this study, PCoA and relative abundance results indicated that feeding CYC can adjust the richness and diversity of the microbial community structure in the jejunum. Specifically, at the species level, there was a notable increase in the abundance of *Bifidobacterium pseudocatenulatum* and *Faecalibacterium prausnitzii*. It has been reported that *B. pseudocatenulatum* alleviates DSS-induced colitis in mice by increasing Muc2 mRNA expression, thereby enhancing intestinal barrier function while reducing pro-inflammatory cytokines TNF-α, IL-1β, and IL-6, and increasing the anti-inflammatory cytokine IL-10 (49), indicating the anti-inflammatory and immune-regulatory benefits of *B. pseudocatenulatum* for the gut. *F. prausnitzii* is a major producer of butyrate in the intestine (50), and a reduction in *F. prausnitzii* has been negatively correlated with disease activity (51). Butyrate is reported to be a primary energy source for intestinal epithelial cells (IEC), which are essential for maintaining barrier integrity (52). Therefore, butyrate plays a crucial role in preserving the integrity of the intestinal barrier. Research has shown that butyrate and its derivatives can reduce intestinal inflammation by promoting mucus secretion (53), and effectively enhance gut development (54). In this study, feeding CYC resulted in an increased abundance of *B. pseudocatenulatum* and *F. prausnitzii*, which elevated the expression of Muc2 and reduced the levels of pro-inflammatory cytokines IL-1β and TNF-α. These observations may be related to the effects of butyrate. We conducted a Spearman correlation analysis to examine the relationship between changes in gut microbial community structure and intestinal barrier function, as well as cytokines, to validate the reasoning behind our observations. The study found that *F. prausnitzii* in the intestines of laying hens showed a significant positive correlation with Muc2, occludin, and claudin-1 (*p* < 0.05), while it exhibited a significant negative correlation with IFN-γ and TNF-α (*p* < 0.05). Similarly, *B. pseudocatenulatum* was positively correlated with Muc2, claudin-1, occludin, TGF-β1, IL-10, IL-4, and Zo-1, and negatively correlated with IFN-γ and TNF-α. We hypothesize that *B. pseudocatenulatum* and *F. prausnitzii* have a regulatory effect on the mRNA expression of Muc2, claudin-1, occludin, TGF-β1, IL-10, IL-4, and Zo-1, which aligns with the results mentioned above. These findings further suggest that the complex yeast culture (CYC) may promote butyrate production by increasing the relative abundance of *B. pseudocatenulatum* and *F. prausnitzii*, thereby improving intestinal barrier function and modulating the expression of associated inflammatory cytokines. However, the underlying mechanisms involved require further investigation.

## 5 Conclusion

In conclusion, the complex yeast culture (CYC) may enhance the intestinal microbial community structure in late-stage Hy-Line Brown hens, improve intestinal barrier function, and reduce the occurrence of intestinal inflammation. These effects contribute to increased egg production and alleviation of intestinal inflammation. Therefore, CYC can serve as an effective feed additive to strengthen the intestinal structure of laying hens, promoting better nutrient absorption and immune defense during the later stages of egg production. This study provides a theoretical basis for the clinical application of complex yeast cultures in the poultry industry.

## Data Availability

The datasets presented in this study can be found in online repositories. The names of the repository/repositories and accession number(s) can be found in the article/supplementary material.
